# Medical Statistics

**Published:** 1837-07

**Authors:** 


### MEDICAL STATISTICS.

*On the Statistics of the Negro Slave Population in the West Indies.* By A. M. TULLOCH, Esq.

THIS is a most valuable paper by a very able statistician. It proves a fact, highly important both in a political and medical point of view, namely, that the number of the black population in our western colonies is progressively decreasing and in a most remarkable degree. This decrease is scarcely in any degree owing to deficiency in the number of births, but almost entirely to the increased number of deaths. The mortality in this kingdom is stated by Mr. Rickman to have been one in fifty-one on the average of the five years preceding 1830; but that of the negro population in the West Indies has been one in thirty-six.* Another singular feature in this case is the fact that the ratio of mortality is so very much greater in the male sex than in the female, the proportion in *adults* being nearly *double*

* The island of Jamaica, however, seems to be an exception to the general rule.

among males. It is to this disproportionate mortality of males rather than to the high mortality of the whole population, that the great decrease of the negro population is to be attributed.

Neither is it, as might have been supposed, to any peculiarity in their condition as *slaves* that this great mortality is attributable, but to the same general causes that render the constitutions of one race of men unapt to be assimilated to the climate of other countries. This is a fact quite at variance with our preconceived opinions, and deprives the advocates of slavery of one of the strongest arguments usually addressed to its supporters on political grounds.

"It is an erroneous idea," says Mr. Tulloch, "to suppose that the negro race are exempt from the fatal influence of fever in the West Indies. It may not, indeed, produce the same wide-spreading devastation as among Europeans, but it creates at least thrice as great a mortality as among an equal extent of population in this country. Diseases of the bowels, too, are of very frequent occurrence, manifesting themselves in diarrhœas, and occasionally also in dysenteric affections. Even where these produce not immediate fatal effects, their frequent recurrence is sure to debilitate the frame, and act as an insuperable barrier to the attainment of old age. But of all the diseases which tend to the rapid diminution of this ill-fated race, those of the lungs are by far the most frequent and fatal. Though none of the Parliamentary documents furnish any specific details as to the numbers who die annually among the slave population from this cause, yet all the evidence attributes a large proportion of the deaths to their peculiar liability to this class of diseases, particularly between the ages of thirty and forty-five, which in a great measure accounts for the high ratio of mortality at that period of life. Nor is the peculiar predisposition of the negro to this class of diseases confined to the West Indies, for it manifested itself in a still greater degree among that description of troops when employed in Ceylon, and has also been productive of much mortality among those in the Mauritius.

"Whether succeeding generations will become less susceptible of the diseases which that climate at present induces, and thereby the mortality be sufficiently reduced to enable the births to supply the yearly decrement by deaths, is a problem which time alone can solve; but, if not, there can be little doubt, as the decrease is now proceeding at the rate of a tenth part of the whole population every fourteen years, that before the termination of another century, this race will have almost ceased to exist in our West India colonies; and as there can now be no fresh importations from other quarters, unless nature supplies the great waste of male as compared with female life, we shall, even in the present generation, see all the colonies exhibiting the same remarkable superabundance of females as in the old settlements of Barbadoes and Antigua."

No satisfactory explanation is given of the superior mortality of the males in these islands, which is, as Mr. Tulloch observes, "certainly one of the most striking features ever exhibited in the vital statistics of any race.

"It seems difficult to assign any reason, for this remarkable exemption in favour of the females, more probable than that there may be some peculiarity in the constitution of that sex which renders them more susceptible of assimilation to foreign climates, and thereby less obnoxious to their influence. It has often been remarked that European females suffer less by a transition to, or residence in, tropical climates than males, which has been attributed to their habits, rendering them less subject to exposure; but when we find the same remarkable feature manifesting itself among a race of females who enjoy no such exemption from exposure, we are led to attribute it to some more general cause."

*British Annals of Medicine.* No. XIII. and XV.

---

### *Rate of Mortality in Sweden in* 1810-1830.

THE very valuable paper on this subject affording important conclusions in what Mr. Farr terms *Vital Statistics,* we can only refer to: it does not admit of extract.

*British Annals of Medicine. March* 24, 1837.

